# Complications of the nasal dorsum reconstruction using autologous or alloplastic grafts: evidence from systematic review and meta-analysis

**DOI:** 10.1016/j.bjorl.2020.07.001

**Published:** 2020-08-08

**Authors:** Jibril Y. Hudise, Saud A. Aldhabaan, Badi F. Aldosari

**Affiliations:** a Facial Plastic Surgery King Saud University, King Abdulaziz University Hospital, Department of Otorhinolaryngology, Head and Neck Surgery, Riyadh, Saudi Arabia; b King Faisal Medical City of Southern Region, Abha, Saudi Arabia

**Keywords:** Complication, Nasal dorsum, Reconstruction, Autologous, Alloplastic grafts

## Abstract

**Introduction:**

Augmentation rhinoplasty depends mainly on intact stable bony and cartilaginous parts. Many trials have used different materials as a graft to perform the operation and support the nose. Debate exists whether alloplastic or autogenic grafts are more appropriate. Common available alloplastic grafts include silicone, medpor, and gore-tex. Autogenic grafts are usually derived from costal cartilages. Warping, infection, and hypertrophic scars are the main complications of the procedure. Yet no subgroup analysis has been performed to investigate the effect of different risk factors.

**Objective:**

To investigate the effect of different types of grafts and the association of the income level of the country on surgery complications.

**Methods:**

A comprehensive literature search of articles was conducted in PubMed, Cochrane Library, Web of Science, and SCOPUS databases through October 2019. We included articles that used autologous or alloplastic grafts in nasal dorsum reconstruction surgery. We performed subgroup analysis according to the type of graft used, region, and income level of the country. A meta-regression analysis model was carried out from the period of 1999–2018, to study the incidence of these complications over time.

**Results:**

The overall complication rate was 7.1%, which was higher in the alloplastic group (7.8%) than the autogenic group (6.9%). The most common complications were secondary surgery for re-correction (4.1%), infection (2.1%), warping (1.6%), and hypertrophic scars (1.6%). All outcomes were homogeneous (I^2^ < 50%).

**Conclusion:**

Patients with autogenic grafts are less liable to develop complications than their peers reconstructed with alloplastic grafts. Moreover, Asian patients are less susceptible to overall rhinoplasty complications. Attention should be noted for low-income countries in which surgical complications are more prone to occur.

## Introduction

Rhinoplasty is the most common cosmetic surgery. Correction of nasal septal deformity is a challenge for aesthetic surgeons to provide the best shape of both the bony and cartilaginous frameworks of the nose with minimal complications.^1^ Materials for reconstruction include autogenic and alloplastic grafts.^2^ Autogenic transplantation of costal cartilage grafts is characterized by fewer infections, patient safety, and mild immunological response.^3^ However, alloplastic grafts are better in terms of less warping, unlike costal cartilage grafts, which may absorb or warp during the period after surgery.^4^ To overcome these difficulties, several techniques are used to minimize graft-related warping, the most frequent of which is delay grafting or immersing the graft in a solution before shaping or insertion.^5^

Rhinoplasty is becoming more common among patients over 40 years.^1^ For a cosmetic purpose, many women have been seeking for a diversity of beauty patterns. Although autogenic grafts are more commonly used than alloplastic grafts,^6^ alloplastic grafts are widely preferable in Asia over autogenic grafts.^7^

Currently, there is a debate relating to the effect of rhinoplasty on quality of life. A prospective study of Hosseinzadeh et al., 2017 found that cosmetic rhinoplasty improves the quality of life of the patients.^8^ Another study by Zojaji et al. 2014 reported that cosmetic rhinoplasty has no significant effect on general health and quality of life except for its psychological health advantages, independently on sex, age, marital, or educational status.^9^

Complications of rhinoplasty are different according to ethnic and regional factors. Rhinoplasty of the bulbous and saddle nose is a standard aesthetic procedure in Asian countries.^10^ The Asian nose is characterized by tight skin, with weak and scant cartilage. This results in increased frequency of the bulbous tip, small nose, and a short nose. It is not easy to shape straight cartilage from a curved rib.^11^ The conventional way to correct saddle noses is to insert an implant into the subperiosteal pocket, which can lead to a severe postoperative complication.^10^

The systematic review of Varadharajan and his colleagues found that autologous costal cartilage rhinoplasty is associated with significant side effects as pneumothorax, pleural tear, and most commonly, warping.^12^ In another review, Lee et al.,^13^ found that the use of alloplastic materials leads to more extrusion and displacement. These side effects are more evident in the pediatric population; a recent review^14^ found that young children experience more revisions than adults.

A previous retrospective study compared two alloplastic grafts; gore-tex and medpor and found an increase in infection rates in the latter group; however, the topic lacks research about essential factors that may play a role in modifying surgery results. These factors may include ethnic factors, as the Asian nose is more common for rhinoplasty than other non-caucasian noses.^15^ Another factor is the type of graft used, whether alloplastic or autogenic. The income of the country may play a role in the quality of surgery on an individual basis.

Therefore, we present this systematic review and subgroup analyses to investigate the effect of different types of grafts and the income of the country on surgery complications and to compare patients in terms of study setting to explore if the people living in Asia will show different results than people from Europe. We also investigated the time factor from 1999 to 2018 through a meta-regression model to examine if the complications of surgeries have improved over time.

## Methods

### Search strategy and study selection

The study process was conducted following the accepted methodology recommendations of the PRISMA checklist for systematic review and meta-analysis where registration of the protocol is not mandated.^16^ We searched PubMed, Scopus, Web of Science, Virtual Health Library (VHL), Google Scholar, and Cochrane databases. The search was done using the following search strategy “(nasal reconstruction OR rhinoplasty OR nasal augmentation OR nasal reconstruction OR nasal dorsum reconstruction OR nasal plastic surgery) AND (alloplastic implant OR nasal implants silicone OR Medpor OR Gore-Tex) AND (costal OR costal cartilage OR rib OR osteocartilaginous rib graft).

We marked studies as included if they met the following criteria: 1) Study design: Clinical trials: comparative or non-comparative; randomized or not randomized clinical trials; 2) Population: patients who underwent nasal dorsum reconstruction; 3) Intervention: rhinoplasty using autologous graft (autologous costal cartilage) or alloplastic grafts; 4) Outcome: at least one of the surgery-related complications. We excluded studies in the following conditions: 1) Observational or retrospective studies; 2) Studies of homologous rib graft; 3) Animal studies, in vitro studies, review articles, case reports, conference abstracts, and duplicate publications.

### Data extraction

Two authors performed the extraction sheet on Microsoft Excel file by pilot extraction of at least three papers. Three reviewers independently extracted data from included studies using the excel sheet. The fourth reviewer performed data checking for checking the accuracy of the extracted data. All the disagreements and discrepancies were resolved by discussion and consultation with a senior member when necessary.

### Risk of bias assessment

We performed the risk of bias assessment using ROBINS-1 (Risk of bias in non-randomized studies of interventions) tool. It includes the following domains: 1) Bias due to confounding; 2) Bias in selection of participants into the study; 3) Bias in classification of interventions; 4) Bias due to deviations from intended interventions; 5) Bias due to missing data; 6) Bias in the measurement of outcomes, and 7) Bias in the selection of the reported result. Studies are marked to either low, moderate, or high risk of bias according to each domain.

### Statistical analysis

We used OpenMeta [Analyst] Software (Center of Evidence-Based Medicine, Brown University, School of Public Health, Rhode Island State, USA) to perform the meta-analysis of all outcomes. The mean proportions of complication rates were pooled in a meta-analysis model, using the Mantel-Haenszel method. The analysis was performed under the fixed-effects model for homogeneous data, and the random-effects model for heterogeneous data. The corresponding 95% Confidence Intervals (95% CI) of pooled effect size were calculated using a random-effects due to the presence of heterogeneity.

Heterogeneity was assessed with Q statistics and I^2^ test considering it significant with I^2^ value > 50%.^17^ Publication bias could not be assessed using Egger’s regression test due to the small number of included studies (less than 10).^18^, ^19^ The publication bias was assessed using Egger’s regression test^18^, ^19^ and represented graphically by Begg’s funnel plot^20^ when there were ten or more studies. Egger’s regression test *p*-value <0.10 was considered significant. Whenever publication bias was found, the trim and fill method of Duvall and Tweedie was applied to add studies that appeared to be missing^21^ to enhance the symmetry. A meta-regression model was done to correlate the time of surgery and the occurrence of different complications.

## Results

### Results of the literature search

**Figure 1:**
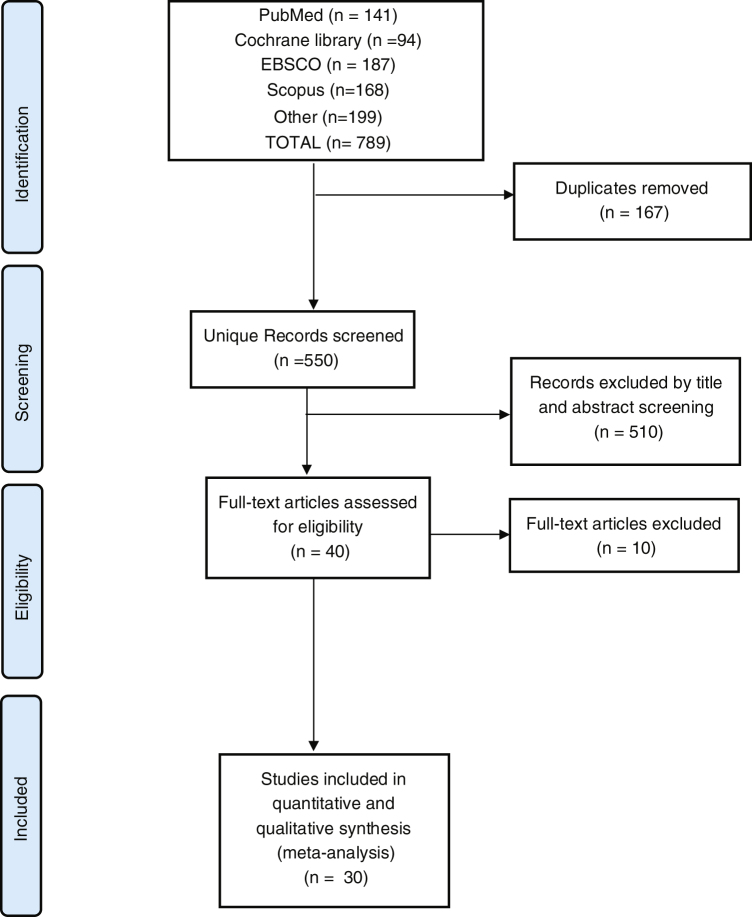
PRISMA flow diagram.

Our search yielded a total of 789 studies. Following screening and excluding duplicates, 40 studies remained that entered full-text screening. We excluded 10 studies due to either lack of outcomes or studies of non-human species. Finally, 30 studies were included in the meta-analysis, as reported in the PRISMA flow diagram, Fig. 1.

### Summary of included studies

We included 30 studies, two of which were controlled trials; therefore, we treated each group as a separate study for feasibility in performing a single-arm meta-analysis. Autogenic grafts were used in 21 studies, while 10 studies used alloplastic grafts. One study used a combination of alloplastic and autogenic grafts.^22^ A total of 1013 patients were enrolled in this meta-analysis, with a mean age of 27.7 years, and a mean duration of follow-up after surgery of 22 months. Table 1 shows a summary of baseline characteristics for each study.

**Table 1:** Summary of included studies.

Study and year	Country	Surgery type	Type of graft	Sample size	Males, n (%)	Mean duration of follow-up, months	Randomization	Mean age, years
Agrawel 2015	India	NR	Autogenic	51	15 (29.41%)	26	No	NR
Al-Qattan 2007	Saudi Arabia	Open	Autogenic	21	3 (14.29%)	48	No	31
Baek 2010	Korea	Open	Autogenic	28	17 (60.71%)	12	No	NR
Cakmak 2002	Turkey	Open	Autogenic	20	12 (60.00%)	20	No	30
Cerkes and Basran 2016	Turkey	Open	Autogenic	109	46 (42.20%)	19.6	No	27
Draf 2003	Germany	Combined Approach	Autogenic	10	NR	7.5	No	17
Gu 2018	China	Closed	Alloplastic	76	4 (5.26%)	106	Yes	31
Gurlek 2006	Turkey	Open	Alloplastic	15	NR	16	No	32
Jiang 2013	China	Closed	Alloplastic	19	15 (78.95%)	10.5	No	32.2
Karaaltin 2012	Turkey	Open	Autogenic	23	14 (60.87%)	24.5	No	29
Kayabasoglu 2015	Turkey	NR	Autogenic	23	NR	16	No	34
Khorasani 2018	Iran	NR	Alloplastic	16	3 (18.75%)	6	No	26.3
Kim 2011	Korea	NR	Alloplastic	38	7 (18.42%)	2.5	No	28.5
Kim and Kim 2013	South Korea	Open	Autogenic	58	40 (68.97%)	5.5	No	NR
Lee 2018	Korea	NR	Alloplastic	12	4 (33.33%)	22.25	No	24.5
Lee and Ham 1999	Korea	Closed	Autogenic	47	NR	2.5	No	6.5
Li 2010	China	Open	Autogenic	26	NR	9.6	No	29
Li 2018	China	Open	Alloplastic	17	12 (70.59%)	18	No	NR
Lin 2006	China	Open	Autogenic	19	3 (15.79%)	20	No	
Ma 2015	China	Closed	Autogenic	22	NR	8.9	No	26.8
Riechelmann 2004	Germany	Open	Autogenic	43	11 (25.58%)	24	No	36
Shubailat 2003	Jordan	Open	Autogenic	47	13 (27.66%)	96	No	NR
Swaroop 2018	India	Open	Autogenic	10	15 (29.41%)	6.5	No	NR
Tastan 2013	Turkey	Closed	Autogenic	69	3 (14.29%)	NR	No	NR
Tastan-Sozen 2013	Turkey	Closed	Autogenic	43	17 (60.71%)	19.2	No	NR
Turegun 1998	Turkey	Closed	Alloplastic	14	12 (60.00%)	30	No	NR
Turegun 2008	Turkey	Closed	Alloplastic	36	46 (42.20%)	14	No	NR
Wang 2013	China	Open	Combined Autogenic and Alloplastic	46	NR	10	No	34
Yazar 2015	Turkey	Open	Autogenic	17	4 (5.26%)	19	No	24
Yilmaz 2007	Turkey	NR	Autogenic	38	NR	27.4	No	NR

NR, not reported.

### Results of risk of bias assessment

We reported an overall low risk of bias according to the ROBINS-1 tool. All studies had a low risk of confounding bias, the bias in classification of interventions, bias due to deviations from intended interventions, the bias in the measurement of outcomes, and bias in the selection of the reported result. Nine studies^23^, ^24^, ^25^, ^26^, ^27^, ^28^, ^29^, ^30^, ^31^ did not report enough baseline data about patients such as sex and mean age. Therefore, these studies marked a moderate risk of bias.

**Figure 2:**
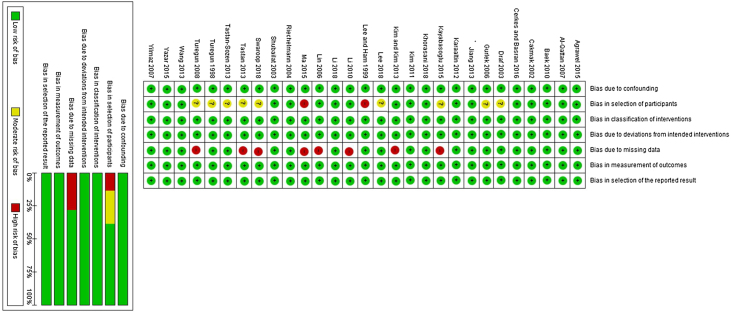
Summary of risk of bias for included trials using ROBINS-1 tool.

Regarding missing data, eight studies^25^, ^27^, ^28^, ^30^^,^^32^, ^33^, ^34^ did not report necessary outcome endpoints to be included in a meta-analysis. We did not find any data for important complications of the surgery such as infection, therefore these studies were put to a high risk of bias. The risk of bias summary was of included studies was reported in Fig. 2.

### Overall complication rates

**Figure 3:**
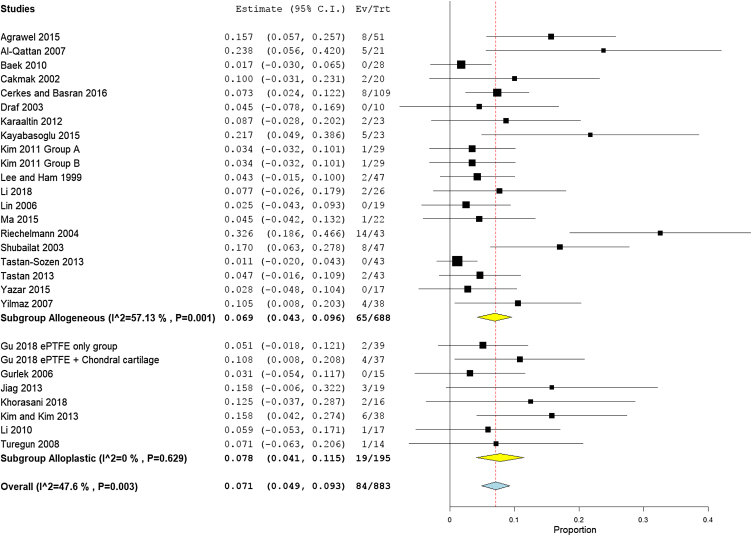
Forest plot of total complications rate.

The overall complication rate was 7.1% (95% CI 4.9%−9.3%) which was higher in the alloplastic grafts group 7.8%, (95% CI 4.1%−11.5%) than the autogenic group 6.9%, (95% CI 4.3%− 9.6%). There was no heterogeneity among the included studies (I^2^ = 47.6%) (Fig. 3). One study^22^ used combined alloplastic and autogenic graft, therefore, it removed from the subgroup analysis by graft type.

### Prevalence of warping

**Figure 4:**
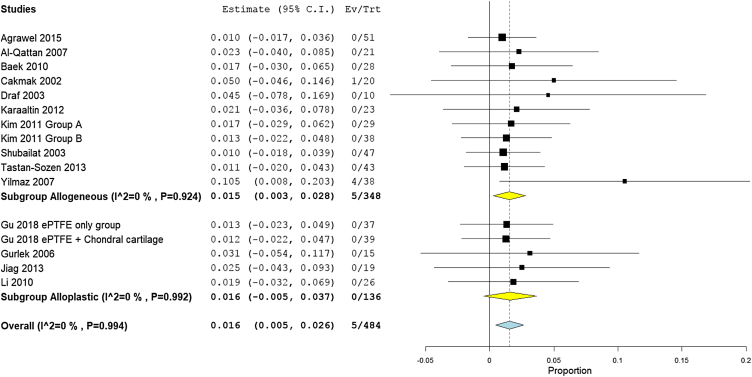
Forest plot of warping outcome according to graft type.

The overall warping rate as reported by fifteen studies (530 participants) was 1.6% (95% CI 0.5%−2.6%). Four studies^16^, ^24^, ^35^, ^36^ used alloplastic grafts and reported a prevalence rate of 1.6% (95% CI −0.5% to 3.7%), while in the autogenic group, the pooled proportion of warping as reported by ten studies^18^, ^19^, ^20^, ^21^, ^23^, ^28^, ^33^, ^37^, ^38^, ^39^ was 1.5% (95% CI 0.3%−2.8%) (Fig. 4).

**Figure 5:**
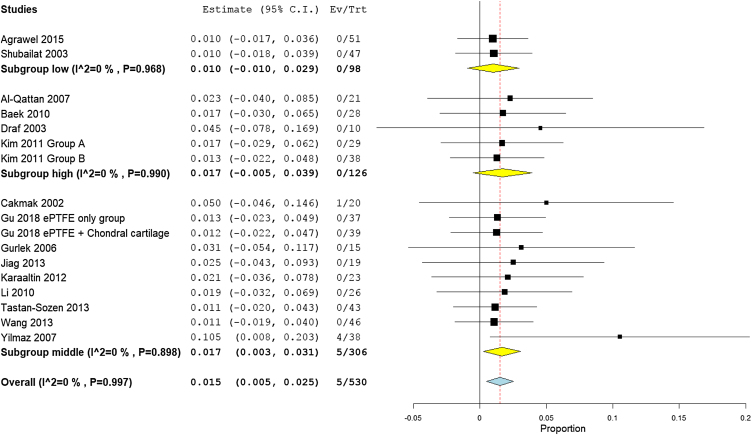
Forest plot of warping outcome according to country income level.

**Figure 6:**
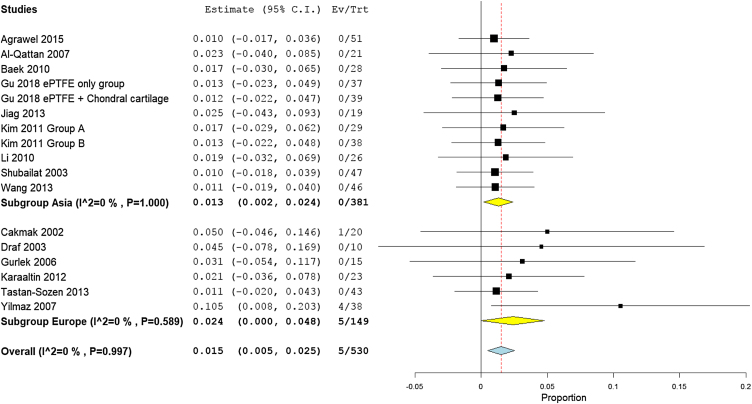
Forest plot of warping outcome according to the study sitting.

Pooling the results from four studies of high-income countries,^21^, ^23^, ^33^, ^37^ nine studies of middle-income countries,^16^, ^17^, ^18^, ^19^, ^22^, ^24^, ^28^, ^35^, ^36^ and two low income studies revealed a prevalence rate of 1.7% (95% CI −0.5% to 3.9%), 1.7% (95% CI 0.3%−3.1%), and 1.0% (95% CI −1.0% to 2.9%), respectively (Fig. 5). Similarly, the mean proportion of warping rate from Asian studies^16^, ^20^, ^21^, ^22^, ^33^, ^35^, ^36^, ^37^, ^38^ was 1.3% (95% CI 0.2%−2.4%), while in European studies^16^, ^17^, ^18^, ^19^, ^23^, ^24^, ^28^, ^36^ was 2.4% (95% CI 0%−4.8%) (Fig. 6). Pooled studies, in all analyses, did not show any significant heterogeneity.

### Prevalence of infection

**Figure 7:**
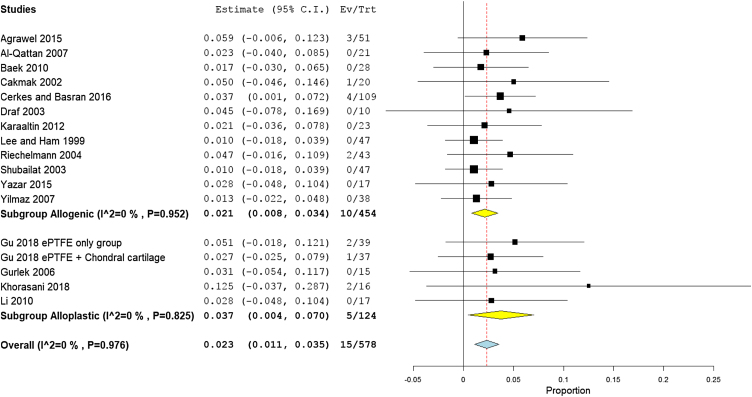
Forest plot of infection outcome according to graft type.

The prevalence of infection was reported by 17 studies (578 patients). The overall infection rate of the nasal dorsum reconstruction surgery was 2.1% (95% CI 1.0%−3.3%). The alloplastic grafts were associated with higher infection rates of 3.7% (95% CI 0.4%−7.0%), compared to only 2.1% (95% CI 0.8%−3.4%) among autogenic grafts (Fig.7).

**Figure 8:**
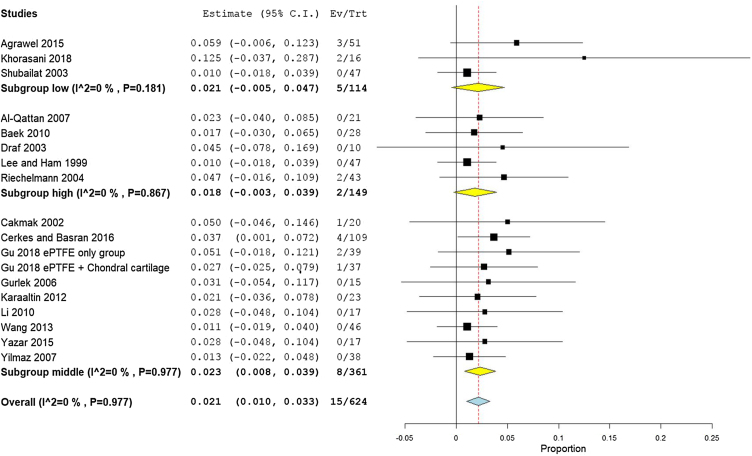
Forest plot of infection outcome according to country income level.

**Figure 9:**
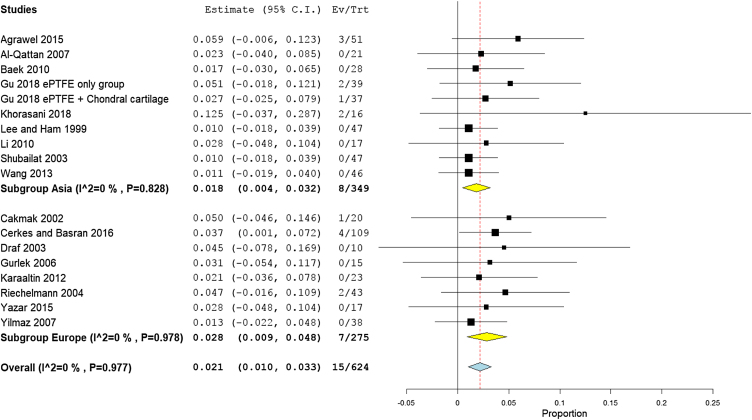
Forest plot of infection outcome according to study sitting.

Rates of infection were lowest in high income countries with 1.8% (95% CI −0.3% to 3.9%), followed by middle, and low income countries, with 2.3% (95% CI 1%−3.3%), and 2.1% (95% CI −0.5% to 4.7%), respectively (Fig. 8). The results showed that according to patients’ regional country, nine Asian studies^16^, ^20^, ^21^, ^22^, ^35^, ^37^, ^38^, ^40^, ^41^ showed lower infection rates of 1.8% (95% CI 0.4%−3.2%), compared to Eight European studies^17^, ^18^, ^19^, ^23^, ^24^, ^39^, ^42^, ^43^ that showed an infection rate of 2.8% (95% CI 0.9%−4.8%) (Fig. 9). Pooled studies, in all analyses, did not show any significant heterogeneity.

### Hypertrophic scar

**Figure 10:**
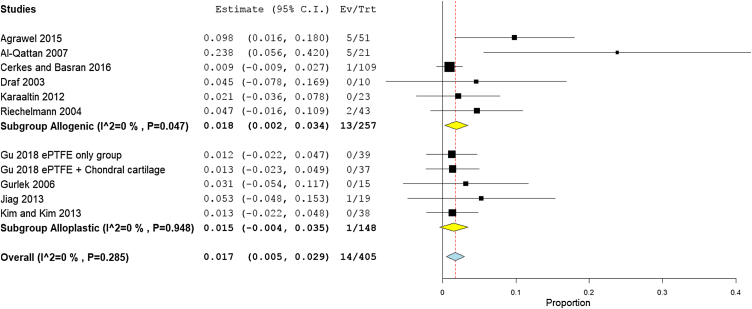
Forest plot of hypertrophic scars outcome according to graft type.

Eleven studies (405 patients) reported the prevalence rates of all scars. The overall pooled rate was 1.7% (95% CI 0.5%−2.9%), for both nose and chest scars. Subgroup analysis showed that prevalence of scars in autogenic grafts reported by six studies^18^, ^20^, ^23^, ^37^, ^39^, ^42^ was 1.8% (95% CI 0.2%−3.4%), while in the alloplastic grafts, reported by four studies,^24^, ^35^, ^36^, ^44^ the scarring rate was 1.5% (95% CI −0.4% to 3.5%) (Fig. 10).

**Figure 11:**
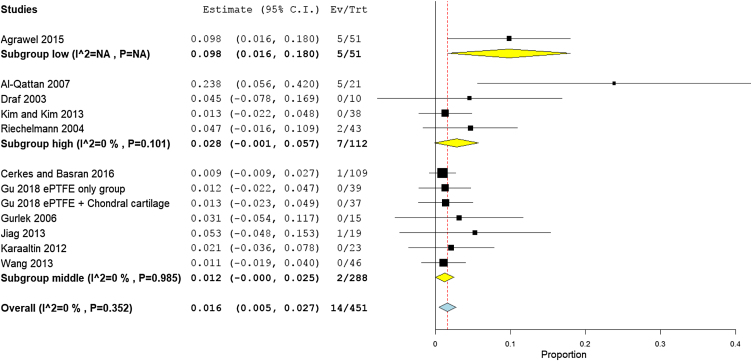
Forest plot of hypertrophic scars outcome according to country income level.

**Figure 12:**
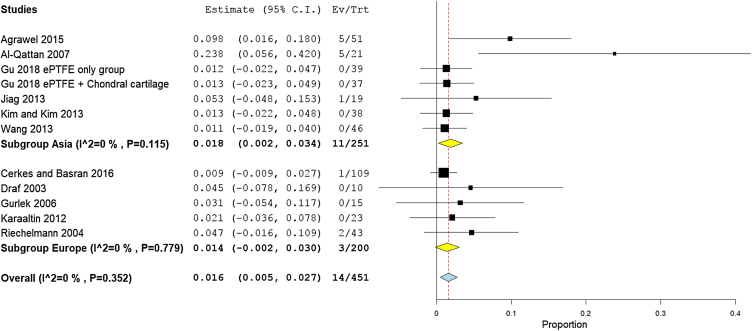
Forest plot of hypertrophic scars outcome according to study sitting.

Stratifying studies according to countries, the scarring rate was the highest in low income countries with 9.8% (95% CI 1.6%−18%), followed by high income countries with 2.8% (95% CI −0.1% to 5.7%), and middle-income countries with 1.2% (95% CI −0% to 2.5%) (Fig. 11). The Asian studies reported higher scarring rates of 1.8% (95% CI 0.2%−3.4%) as reported in six studies.^20^, ^22^, ^35^, ^36^, ^39^, ^44^ European patients experienced 1.4% scar rates (95% CI −0.2% to 4.8%) as reported in five studies^18^, ^23^, ^24^, ^39^, ^42^ (Fig. 12). Pooled studies, in all analyses, did not show any significant heterogeneity.

### Need for a second surgery for re-correction

**Figure 13:**
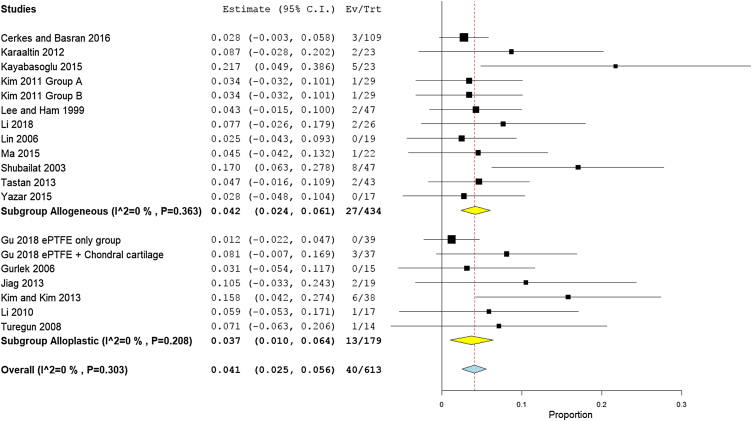
Forest plot of need for a second surgery outcome according to graft type.

A total of 19 studies (613 participants) reported the need for a second surgery. The overall rate of re-correction surgery was 4.1% (95% CI 2.5%−5.6%). Subgroup analysis showed that the need for a second surgery was higher in the autogenic grafts with 4.2% (95% CI 2.4%−6.1%), compared to the alloplastic grafts with 3.7% (95% CI 1.0%−6.4%) (Fig. 13).

**Figure 14:**
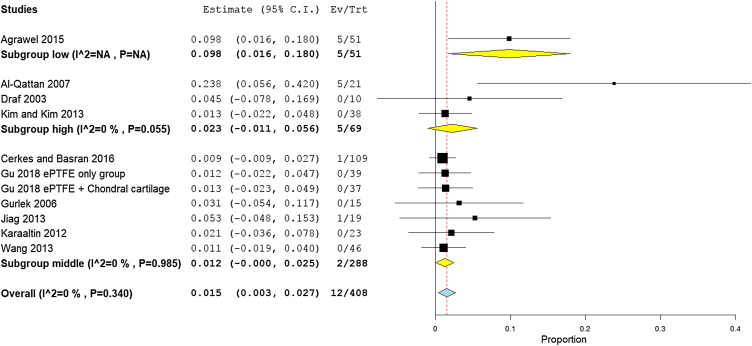
Forest plot of need for a second surgery outcome according to country income level.

**Figure 15:**
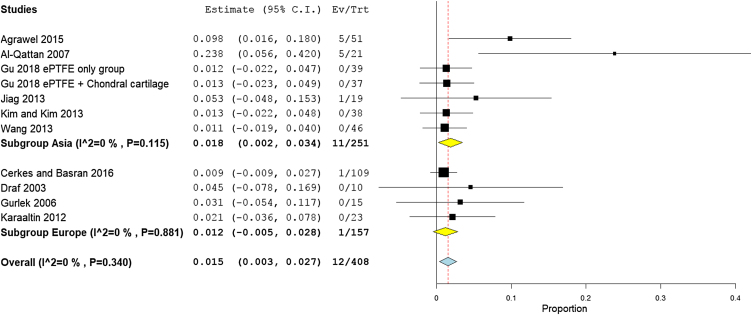
Forest plot of need for a second surgery outcome according to study sitting.

The middle-income countries reported the lowest need for second surgery with only 1.2% (95% CI 0%−25%),^16^, ^18^, ^22^, ^24^, ^25^, ^29^, ^31^, ^32^, ^34^, ^35^, ^36^, ^39^, ^43^, ^45^ followed by high income country studies^33^, ^40^, ^42^, ^44^ 2.3% (95% CI 2.8%−9.5%), and low income countries 9.8% (95% CI 6.3%−27.8%), respectively (Fig. 14). Asian studies reported second surgery rates of 1.8% (95% CI 0.2%−3.4%) as reported by 11 studies,^16^, ^32^, ^22^, ^33^, ^35^, ^36^, ^38^, ^40^, ^44^, ^45^ while a lower rate was observed in the European studies 1.5% (95% CI 0.3%−2.7%) as reported by eight studies^18^, ^24^, ^25^, ^29^, ^31^, ^39^, ^42^, ^43^ (Fig. 15). Pooled studies, in all analyses, did not show any significant heterogeneity.

### Meta-regression

**Figure 16:**
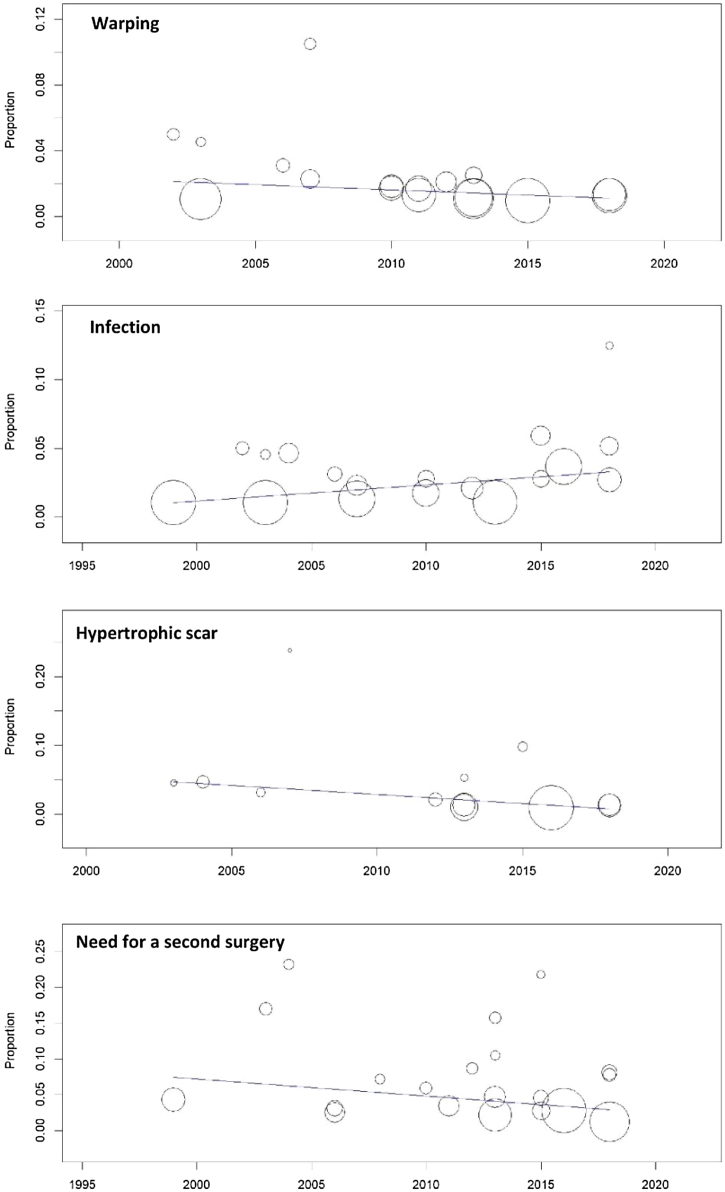
Meta-regression analysis.

A meta-regression model was performed to correlate the date of surgery (from 1999 to 2018) with the occurrence of complications. We found no significant effect of surgery date on warping rates (*p* = 0.6), infection rates (*p* = 0.2), hypertrophic scars (*p* = 0.1), or the need for a second surgery showed (*p* = 0.1) (Fig. 16).

### Publication bias

**Figure 17:**
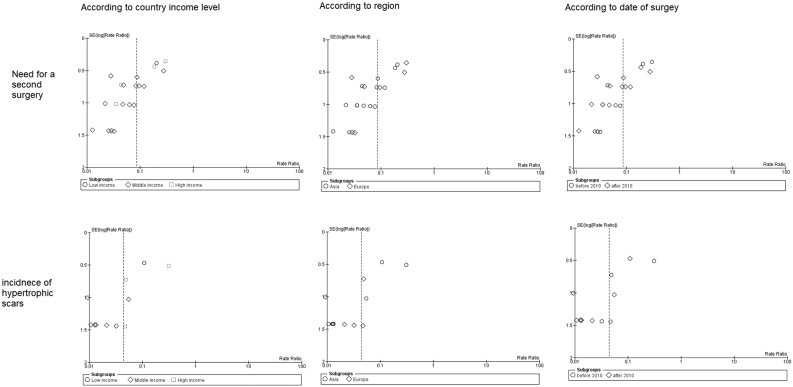
Begg’s funnel plot shows significant publication bias.

**Figure 18:**
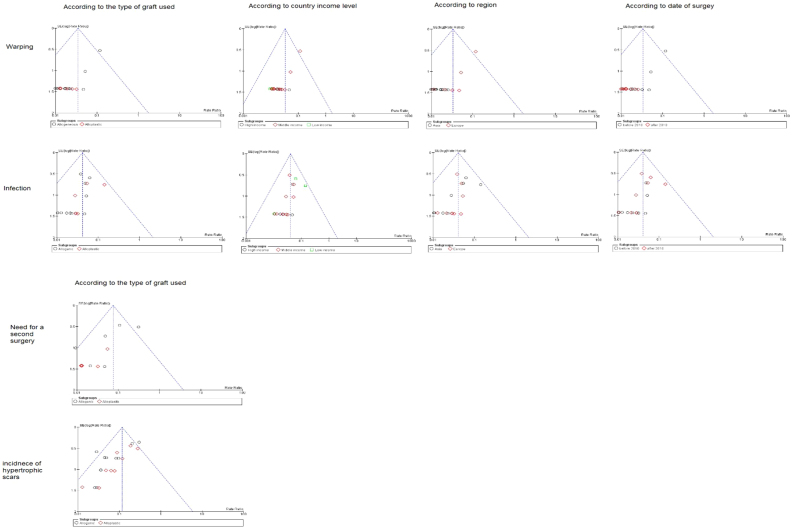
Begg’s funnel plot shows non-significant publication bias.

This meta-analysis reported a significant publication bias in two outcomes: the need for a second surgery and hypertrophic scars (Fig. 17). The rest of the outcomes and subgroup comparisons did not show any similar funnel plot asymmetry, hence no risk of bias (Fig. 18).

## Discussion

The present meta-analysis provides class 1 scientific evidence that the overall complications rate is less common in patients from Asia than in people living in Europe and that autogenic grafts have lower prevalence rates of overall complications. Moreover, patients from low income countries showed increased rates of infection, warping, and need for reoperation, in contrast to high income and middle income countries.

The literature is full of studies that support our results. A previous meta-analysis which included 10 studies found that alloplastic grafts are associated with an increased incidence of infection, warping, hypertrophic chest scarring, and resorption.^44^ They reported that the low number of included studies was their main limitation. In our study, we reported the results from 30 studies (1013 patients) and revealed that the rate of infection, warping, hypertrophic chest scarring, and resorption were 2.1%, 1.5%, 1.6%, and 4.1%, respectively.

Another meta-analysis of observational studies compared alloplastic with autogenic grafts in terms of complications. The study found that autogenic grafts carry the most benefits and least side effects.^46^ This supports our findings; we reported that the alloplastic grafts have a higher infection rate, compared to autogenic grafts (2.1%). Besides, the autogenic grafts require more frequent second surgeries (4.2%) than alloplastic grafts (3.7%).

Our results revealed a high rate of second surgery in patients from Asia; this is supported by the efforts of Matory and Falces^15^ in their research in 1986, where they show that Asian individuals have a weak osteocartilaginous framework that does not easily support grafting. A meta-analysis included autologous costal cartilage grafts showed that rhinoplasty is significantly associated with the incidence of pneumothorax, extrusion, seroma, and persistent donor site pain.^12^ In fact, from the 30 included trials, only two studies reported pain as a significant side effect. Lee et al. reported that synthetic silicone grafts show higher rates of complications.^13^

As expected, low income countries suffer from significant complications of surgery. Consistent with previous literature, middle income countries, that usually do not suffer from such complications,^47^, ^48^ showed less complications compared to low income ones.

The age of patients plays an important role in the incidence of complications because the procedure is more difficult in the pediatric population than adults. Gupta et al. reported that pediatric rhinoplasty is associated with increased revision rates than adults.^14^

Several limitations were observed in the previous meta-analyses: the relatively low number of included trials, lacked subgroup analysis to isolate different factors, low evidence of included studies, and significant heterogeneous outcomes. We considered these critical limitations during the preparation of our meta-analysis.

Our study has many strong points, mainly derived from the limitations of previous works. We performed a comprehensive search that included a large number of studies to provide stronger evidence. Additionally, we included only clinical trials for this meta-analysis. The analyzed data were homogeneous as assessed by I^2^ test. Finally, we added new evidence through our subgroup analyses and meta-regression.

The main limitation we encountered was a lack of data in some studies. Many included studies lacked reporting of important outcomes as warping and infection. Other studies provided data in a manner not suitable for entering in a meta-analysis. The low sample size for each study is an important limitation that should be taken into consideration as well. The studies from low income countries were limited so, it may bias the results and gives underestimation of the actual rates. We recommend future trials with large sample size to adequately assess all relevant outcomes.

In conclusion, patients with autogenic grafts are less liable to develop complications, when compared to their peers with alloplastic grafts. Moreover, Asian patients are less susceptible to overall rhinoplasty complications. Attention should be noted to the fact that in low income countries, surgical complications are more prone to occur.

## Conflicts of interest

The authors declare no conflicts of interest.

## Acknowledgement

We would like to thank King Saud university, College of Medicine. and King Faisal Medical City of Southern Region for their great support of the scientific researches.
